# Perilipins: a diversity of intracellular lipid droplet proteins

**DOI:** 10.1186/s12944-017-0473-y

**Published:** 2017-04-28

**Authors:** Hiroyuki Itabe, Tomohiro Yamaguchi, Satomi Nimura, Naoko Sasabe

**Affiliations:** 1 Division of Biological Chemistry, Department of Molecular Biology, Showa University School of Pharmacy, 1-5-8 Hatanodai, Shinagawa, Tokyo, 142-8555 Japan; 2 Department of Hospital Pharmaceutics, Showa University School of Pharmacy, 1-5-8 Hatanodai, Shinagawa, Tokyo, 142-8555 Japan; 3 Present address: College of Pharmacy, Kinjo Gakuin University, 2-1723 Omori, Moriyaka-ku, Nagoya, 463-8521 Japan

**Keywords:** Perilipin, Lipid droplet, Hydroxysteroid dehydrogenase

## Abstract

Intracellular lipid droplets (LDs) are found in a wide variety of cell types and have been recognized as organelles with unique spherical structures. Although LDs are not stable lipid-depots, they are active sites of neutral lipid metabolism, and comprise neutral lipid or cholesterol cores surrounded by phospholipid monolayers containing specialized proteins. However, sizes and protein compositions vary between cell and tissue types. Proteins of the perilipin family have been associated with surfaces of LDs and all carry a conserved 11-mer repeat motif. Accumulating evidence indicates that all perilipins are involved in LD formation and that all play roles in LD function under differing conditions. In this brief review, we summarize current knowledge of the roles of perilipins and lipid metabolizing enzymes in a variety of mammalian cell types.

## Background

Triacylglycerol (TG) and cholesterol ester (CE) are the major neutral lipids in human bodies. Because of their hydrophobic nature, neutral lipids are insoluble in water and spontaneously form aggregates to minimize lipid–water interfaces. Accordingly, lipid droplet (LD) structures comprise a neutral lipid core surrounded by a monolayer of phospholipids and specialized LD-associated surface proteins. Previous proteomic analyzes have demonstrated distinct protein profiles of LDs from various sources [1–5]. Moreover, certain LD proteins have been distinctly localized on LD particles in immunocytochemical analyzes [6–8], and phospholipid compositions of LD reportedly differ from those of hepatic endoplasmic reticulum (ER) membranes [9]. These observations have led to wide acceptance of LDs as unique organelles [10].

Although intracellular LDs are reportedly ubiquitous, their neutral lipid contents vary between cell and tissue types. In particular, unilocular LDs occupy considerable portions of the cytosol in white adipose tissues (WAT), which are sites of fat storage in mammals. Although the liver is a major lipid metabolizing organ, hepatic LDs are small and are present in limited numbers under physiological conditions. However, excessive lipid accumulation in fatty livers is associated with increased numbers and sizes of cytosolic LDs. In atherosclerotic lesions, macrophage-derived foam cells accumulate large amounts of TG and CE from lipoproteins. Moreover, specialized hormone-secreting cells contain CE storage LDs from which steroid hormones are produced.

Over the last two decades, LDs have been extensively characterized in terms of size, lipid content, and protein composition. The perilipin proteins PLINs1–5 are the major LD-associated proteins (Table 1) [11, 12], and whereas PLIN1 is predominantly expressed in adipocytes, PLIN2 is expressed ubiquitously. Because PLIN1 and PLIN2 are located on LD particle surfaces, they are considered marker proteins for LDs. Recent studies on other PLIN proteins suggest roles in the formation of LDs, and both diversity and redundancy have been demonstrated among them. In addition, some lipid metabolizing enzymes reportedly act as major LD components in certain cell types. In this brief review, we summarize the characteristics of major LD proteins, including PLIN proteins.

**Table 1:** Basic characteristics of PLIN proteins

Proteins	Alternative names	Major cite of expression	Other cites of expression	Function
PLIN1	Perilipin A	WAT	BAT, cardiac muscle liposarcoma	hormone-induced lipolysis large LD stabilization
PLIN2	ADRP, ADFP (human ADRP) Adipophilin	Liver	premature adipocytes macrophages sebocytes mammary gland epithelia choriocaricinoma cells (BeWo) ubiquitously expressed	adipocyte differentiation small LD generation LD stabilization
PLIN3	TIP47	ubiquitous	skeletal muscle neutrophils, mast cells retinal pigment epithelium sebocytes	LD stabilization (compensation of PLIN2) PGE_2_ production intracellular trafficking
PLIN4	S3–12	WAT	hMSC (induced during differentiation) skeletal muscle	human adipocyte differentiation
PLIN5	MLDP, OXPAT, LSDP5	cardiac muscle BAT skeletal muscle	islet β-cells hepatic stellate cells	LD stabilization FA supply to mitochondria

## Discovery of PLIN proteins

**Fig. 1:**
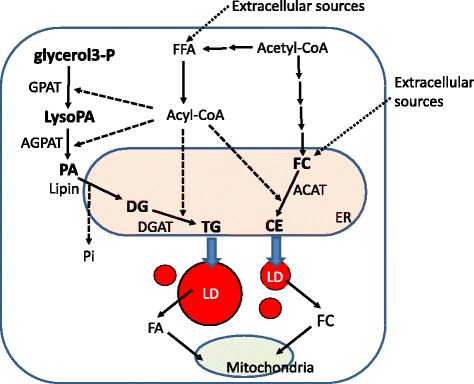
Synthetic pathways of neutral lipids. Triglycerides (TG) are synthesized from one glycerol 3-phophate and three fatty acids. The monoglyceride (MG)-pathway is an alternative TG synthetic pathway in the small intestine, which is not detailed in this figure. Phosphatidic acid (PA) is formed from glycerol 3-phophate by the addition of two acyl chains, and it is subsequently dephosphorylated by lipin to form diglycerides (DG), to which diacylglycerol acyltransferase (DGAT) finally adds a third acyl chain to generate TG. Free cholesterol (FC) is synthesized *de novo* or from acetyl-CoA, or it is derived from extracellular lipoproteins. CE is formed by acylation of FC by acyl-CoA cholesterol acyltransferase (ACAT). These neutral lipids are sources of LDs, and the TG and CE synthetic enzymes DGAT and ACAT are localized in the ER. Neutral lipids accumulate in LDs and they are hydrolyzed for ATP synthesis or steroid hormone synthesis in the mitochondria

Neutral lipids are ubiquitously present in mammalian cells, and their synthetic routes have been investigated previously, as schematized in Fig. 1. TG are synthesized from glycerol 3-phophate and three fatty acids. Specifically, sequential acylation of glycerol 3-phophate produces phosphatidic acid, which is a branch point for the synthesis of TG and phospholipids. Phosphatidic acid is subsequently dephosphorylated by the enzyme lipin, and the resulting diacylglycerol (DG) is converted to TG upon addition of the third fatty acyl group by diacylglycerol acyltransferase (DGAT), which is localized in the endoplasmic reticulum (ER). In contrast, free cholesterol (FC) is either synthesized de novo or is derived from extracellular sources [13], and is acylated by ER localized acyl-CoA cholesterol acyltransferase (ACAT). The neutral lipids TG and CE accumulate in intracellular LDs, and are used as sources of energy or steroid hormone precursors in the mitochondria.

Although the presence of neutral lipid particles in cells has long been known, LDs received little attention until the major phosphorylated protein in adipocytes, perilipin A (PLIN1), was identified on LD surfaces and was shown to regulate the accumulation and hydrolysis of TG [14]. Adipocyte LDs are surrounded by a phospholipid monolayer containing PLIN1, which is phosphorylated by cAMP-dependent protein kinase (PKA) during adrenalin-dependent acute lipolysis. Regulatory mechanisms underlying the formation of LDs in cells have become an active area of research.

**Fig. 2:**
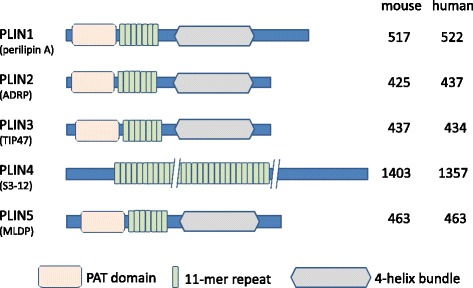
Structures of perilipin proteins. The perilipin family includes 5 members (PLIN1 to PLIN5) with the 11-mer repeat motif, and with the exception of PLIN4, all share a conserved PAT domain. The 4-helix bundle structure is likely present in the C-terminal part of all PLINs except for PLIN4. Numbers of amino acids in human and murine PLINs are indicated

Following identification and cloning of perilipin A, adipose differentiation-related protein (ADRP) and tail-interacting protein of 47 kDa (TIP47) were found to possess a conserved domain called the PAT domain, which was named after the three proteins perilipin A, ADRP, and TIP47 [15]. In addition to the PAT domain, these proteins have an 11-mer repeat motif in common. TIP47 was initially reported to be associated with protein trafficking between the lysosome and Golgi body [16], and S3–12 and myocardial LD proteins (MLDP) were subsequently included in this family [17, 18]. Their localization to LD surfaces was confirmed, although TIP47 and S3–12 were also observed in the cytosol (Fig. 2).

PLIN proteins lack putative transmembrane domains, and it remains unclear how PLINs are associated with LD surfaces. Although the PAT domain contributes to protein associations with LDs, other determinants of LD localization have been suggested, including various other parts of PLIN proteins. Specifically, studies of deletion mutants showed that N- and C-terminal regions and central portions of PLIN1 and PLIN2 are required for LD localization [19, 20]. Another recent study demonstrated that the 11-mer repeat forms amphipathic helices that bind micelles and LDs [21]. Accordingly, various point mutations within the 11-mer repeats of PLIN1–3 changed amphipathic amino acid alignments and abolished LD associations. Eleven-mer repeats have been found in other proteins, including apolipoproteins and the Parkinson’s disease protein α-synuclein [22], which reportedly binds LD-associated proteins in lipid-loaded hippocampal neurons [23]. Moreover, a four-helix bundle structure that resembles that of apolipoprotein E (apoE) was identified in a study of the 3-dimensional structure of C-terminal region of PLIN3 [24], and similar structures were predicted in homology analyses of PLIN1, PLIN2, and PLIN5 [21, 25].

Among PLIN family members, PLIN4 has unique structural characteristics, including the absence of a PAT domain and a polypeptide length of almost three-fold those of other PLIN proteins. However, like PLIN1, PLIN4 is expressed in adipose tissues and it is localized to LDs; its 11-mer repeat is likely crucial for LD associations.

### PLIN1

PLIN1 was originally discovered as a major phosphorylated protein in WAT [6]. PLIN1 is abundantly expressed in mature adipocytes, phosphorylated in a cAMP-dependent manner, and localized to LD surfaces during differentiation of 3T3-L1 adipocytes into lipid-accumulating mature adipocytes. On the other hand, PLIN2 is present in immature preadipocytes that contain lipid-poor small LDs; however, it is replaced by PLIN1 in lipid-rich LD particles during differentiation into mature adipocytes. Hence, PLIN1 may be involved in the formation of large unilocular LDs (often larger than 10 μm) that are a unique feature of WAT [8]. PLIN1 has been detected to some extent in BAT and cardiac muscle in addition to WAT. It is interesting that PLIN1 is expressed in liposarcoma which is the most common soft tissue sarcoma of adults but not in non-lipomatous sarocoma [26].

Previously, PLIN1-knockout (KO) mice were established on a C57BL/6 background and double KO db/db mice exhibited striking phenotypes [27, 28]. These mice were lean, they had microscopically reduced LD sizes in adipose tissues, and exhibited increased glucose tolerance and resistance to diet-induced obesity. In addition, lipolytic activities in the WAT were highly activated in PLIN1-KO mice, preventing accumulation of TG in WAT. These observations suggest essential roles of PLIN1 in LD formation and TG metabolism *in vivo*.

**Fig. 3:**
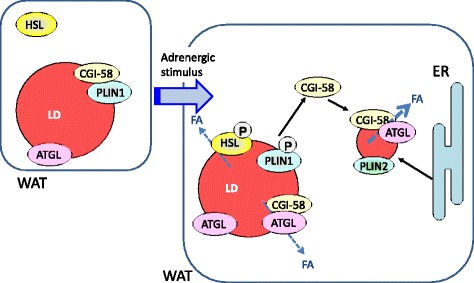
Hormonal regulation of PLIN1-dependent lipolysis in adipocytes. Under unstimulated conditions, PLIN1 is associated with CGI-58, and the PLIN1-CGI-58 complex localizes with ATGL on the surfaces of large LDs. However, ATGL and HSL are inactive under these conditions and lipolysis does not proceed. When the adipocytes are stimulated with adrenergic agonists, hormone sensitive lipase (HSL) and PLIN1 are phosphorylated by activated PKA. Subsequently, phosphorylated PLIN1 releases CGI-58, which binds and activates ATGL. Concurrently, numerous small LDs containing PLIN2 are generated from the ER, and ATGL-CGI-58 complexes localize on the small LDs and actively hydrolyze TG. Phosphorylated HSL also moves to LD surfaces and participate in lipolysis

Lipolysis is activated by hormonal stimulation in WAT and follows hydrolysis of the three ester bonds by sequential actions of adipose triglyceride lipase (ATGL), hormone-sensitive lipase (HSL), and monoglyceride (MG) lipase. ATGL hydrolyzes TG to generate DG and HSL subsequently converts DG to MG by hydrolyzing an ester bond [29]. Finally, MG is hydrolyzed by MG lipase. PLIN1 and HSL are phosphorylated by PKA following activation by interactions of adrenaline and G protein-coupled β receptors (Fig. 3). Under basal conditions, PLIN1 and ATGL are colocalized on LD surfaces and the LD-associated protein comparative gene identification marker protein-58 (CGI-58; also called α/β-hydrolase D5 (ABHD5)) forms a complex with PLIN1 [30]. Upon phosphorylation of PLIN1, CGI-58 is liberated and then binds and activates ATGL [31]. Phosphorylated HSL is also localized to LD surfaces and hydrolyzes DG in concert with ATGL. Thus, PLIN1 acts as a regulator of TG hydrolysis by controlling the ATGL activator CGI-58, and drives dramatic changes in LD sizes of hormone-stimulated adipocytes, in which numerous small LDs (less than 1 μm) were detected in the cytosol [31]. These small LDs are unlikely to be formed from large LDs, and may originate from the ER. However, ATGL–CGI-58 complexes relocate to these small LDs under activated conditions and small LDs are thought to provide a large surface area that enables rapid hydrolysis of lipids.

Although murine 3T3-L1 preadipocytes are widely utilized as the gold standard in adipose cell research, these may differ from human adipose cells under physiological conditions. Recently, human adipose-derived stem cells from mesenchymal or adipose tissues have become commercially available, and Heid et al. showed that brief supplementation of human adipocytes with oleic acid during differentiation leads to the production of LDs of varying sizes [32]. We also prepared mature human adipocytes from these premature cell sources and observed gradual changes in morphology and the development of large multilocular LDs after 3–4 weeks. Human adipocytes require much longer times for differentiation than 3T3-L1 cells, which normally differentiate into adipocytes within 10–14 days. Moreover, PLIN4 was strongly induced during differentiation of human adipocytes as described below, and PLIN1 was located on LDs in cultured human adipocytes [33].

### PLIN2

PLIN2 is well known as the major hepatic LD protein [34]; it has been associated with macrophage-derived foam cells [35], sebocytes [36], and mammalian gland epithelial cells [37], and it is ubiquitously expressed [38]. PLIN2 was originally named adipose differentiation-related protein (ADRP), reflecting its induction during early adipocyte differentiation [39]. However, although PLIN2 is present in premature adipocytes, it is replaced by PLIN1 during differentiation into mature adipocytes [8].

Overexpression of PLIN2 induces LD accumulation in COS-7 cells and fibroblasts [7, 40]. Moreover, PLIN2 knockout mice are resistant to diet-induced obesity, fatty liver disease and alcohol-induced steatosis, suggesting that PLIN2 is responsible for lipid accumulation, particularly in the liver [41, 42]. Taken together, these studies suggest that PLIN2 promotes LD formation and thereby protects TG from lipolysis.

PLIN2 expression is upregulated in many types of cells in which fatty acids are supplied for storage as TG, and is subject to peroxisome proliferator-activated receptor α (PPARα) and γ signaling in various tissues, including the liver and kidney [34, 43, 44]. The other PPAR subtype, PPARδ, also acts as a regulator of PLIN2 expression in some cell types [45, 46]. PPAR sensitive transcription factors hetero-dimerize with retinoid X receptor (RXR), and docosahexaenoic acid (DHA) is reportedly a natural ligand of RXR [47]. Accordingly, we demonstrated that PPARγ and RXR are involved in LD formation as inducers of PLIN2 in BeWo human choriocarcinoma cells [48].

Previous studies indicate that *Plin2* mRNA expression is regulated by PPARs and RXR. However, cellular PLIN2 protein levels are correlated with TG storage levels. BeWo cells accumulate PLIN2-positive LDs following the addition of DHA [49], whereas treatment with a synthetic RXR agonist increased *Plin2* mRNA expression, but not PLIN2 protein levels [10], likely reflecting a balance between PLIN2 translation and degradation. DHA induces *Plin2* mRNA expression and may stabilize PLIN2 protein levels by producing TG, whereas the synthetic RXR agonist may not contribute to TG synthesis. Macrophage-derived foam cells have reduced numbers of LDs and amounts of PLIN2 protein when cultured in the absence of lipid sources. Moreover, the proteasome inhibitor MG132 protected PLIN2 protein levels and led to the accumulation of polyubiquitinated PLIN2 in macrophages [49]. Similarly, ubiquitin-mediated proteolysis of PLIN2 was demonstrated in PLIN2-transfected Chinese hamster ovary cells and mesenchymal embryonic fibroblast (MEF)-derived adipocytes [50, 51]. Hence, whereas PLIN2 stabilizes LD particles in the presence of a high intracellular lipid content, it is likely degraded by the ubiquitin-proteasome pathway under lipid-poor conditions. In a pertinent study, Tomaru et al. investigated the involvement of proteasomal proteolysis of PLIN2 in the regulation of cellular LD accumulation in vivo using a transgenic mouse that overexpressed the proteasome subunit β5t [52]. This transgenic mouse displayed much weaker proteasome chymotrypsin-like activity and severe hepatic lipid accumulation compared with high-fat diet fed wild type mice. While the PLIN2 content was increased in the livers of these transgenic mice, several other LD-associated proteins were unchanged.

Autophagy is an additional mechanism that contributes to LD breakdown via degradation of PLIN2 and TG in hepatocytes [53, 54]. Inhibition of autophagy using the inhibitor 3-methyladenosine, or by deletion of either Atg5 or Lamp2 proteins, accelerated lipid accumulation, suggesting that autophagy is involved in lipid consumption in the liver. Moreover, lipids in LDs were reportedly required for the induction of autophagy in yeast and served as the substrate of autophagosomes [55]. Yeast strains with genetically modified TG or sterol ester synthesis lack LDs, and autophagy is inhibited in these strains. In addition, genes encoding lipases for TG and sterol esters were associated with autophagy. Hence the processes underlying the balance of synthesis, degradation, and storage of intracellular lipids may be mutually regulated.

During maturation of adipocytes PLIN2 is replaced by PLIN1, however mRNA level of *plin2* mRNA remains high and proteasomal degradation of PLIN2 protein is active in the cells. Takahashi et al. reported that the 2nd and 3rd alanine-residues were required to induce ubiquitination and following degradation [51]. Cytosolic PLIN2 was susceptible to degradation but association with LD stabilized PLIN2 in MEF-derived adipocytes.

### PLIN3

Although PLIN3 and PLIN2 have similar amino acid sequences (Fig. 2) and they are both ubiquitously distributed among tissues, it is PLIN3 that localizes to the cytosol and LDs [56]. This protein was initially named “tail-interacting protein of 47 kDa (TIP47)” and it was implicated in intracellular trafficking of lysosomal enzymes [16]. Transport of lysosomal enzymes from the trans-Golgi network to the lysosome is regulated by modifications of oligosaccharide chains and subsequent binding to the mannose-6-phosphate receptor (M6PR). TIP47 binds to the cytosolic domain of M6PR, which leads to its inclusion in transport vesicles. However, no roles of LDs in lysosomal targeting have been reported to date.

Hickenbottom et al. elucidated a four-helix bundle structure that is characteristic of apoE in the C-terminal region of PLIN3 using X-ray crystallography [24]. ApoE and apoA1 are major high-density lipoprotein (HDL) components that detach from lipoproteins and move between particles, and these molecules contain biphasic α-helices that interact with lipoprotein particle surfaces [57]. In addition, a deep cleft is formed by the four-helix bundle in PLIN3 that likely contributes to fatty acid binding and LD recruitment [58]. Similar four-helix bundle structures were also predicted in homology searches of PLIN1, PLIN2, and PLIN5 [21, 25]. In addition to four-helix bundles, lipid-binding cleft formed in the middle part of PLIN2 was proposed by Najt, et al. [59]. They found there are critical hydrophobic amino acid residues that are conserved between PLIN3 and PLIN2.

Knockdown of PLIN3 using siRNA led to decreased TG levels in THP-1 macrophages. In addition, expression of full length PLIN3 resulted in TG accumulation in THP-1 cells [60]**,** and compensatory upregulation of PLIN3 was reported in a PLIN2-null embryonic cell line [61]. PLIN3 binds to sites of LD formation when DG accumulates in the ER membrane [62]. Consistently, recruitment of PLIN3 to LDs was facilitated upon generation of all-*trans*-retinoic acid following photobleaching in the retinal pigment epithelium [63]. Despite its distribution in the cytosol and LDs, previous reports suggest that PLIN3 contributes to the formation and stabilization of LDs. Patel et al. identified functional similarities between PLIN3 and PLIN2, in comparison with PLIN1 [64], and whereas the C-terminal domain of PLIN1 binds and stabilizes CGI-58 and protects LDs from lipolysis, PLIN2 and PLIN3 cannot bind CGI-58 and facilitate higher basal lipolysis rates. Several studies have reported co-expression of PLIN2 and PLIN3 and compensatory activities of PLIN3 in many tissues, although distinct functions of PLIN3 were unknown until recently.

Small lipid-rich vesicles were identified in neutrophils as osmium-sensitive particles in electron microscopy analyses from the early 1970’s [65]. In leukocytes, these vesicles were referred to as lipid bodies, and were later described as a type of LD. LDs in leukocytes are induced by inflammatory stimuli, and LD formation was reportedly involved in eicosanoid generation [66]. Inflammatory mast cells also accumulate lipid bodies in the cytosol during maturation, and Dichlberger et al. showed the presence of PLIN2- and PLIN3-coated LD particles in human mast cells and in cultured LAD2 mast cells [67].

Immortalized HL-60 cells originated from a human promyeloleukemia, and can be differentiated into neutrophils or macrophages under specific culture conditions. Lipopolysaccharide (LPS) treated HL-60-derived neutrophils secreted PGE_2_, accumulated TG, and formed multiple small LDs. Among PLIN family proteins, PLIN3 was predominantly induced by LPS treatment and immunocytochemical analyses showed localization to LDs in HL-60-derived neutrophils [68]. Knockdown of PLIN3 using siRNA inhibited the formation of LDs and the secretion of PGE_2_. These results suggest that PLIN3 plays a crucial role in LD formation and PGE_2_ generation in neutrophils. However, PLIN2 was not detected under these conditions, suggesting that PLIN3 is the predominant LD-associated protein in these cells.

Sebocytes are unique cells of the sebaceous gland and secrete sebum, which is a mixture of TG, wax, squalene, and free fatty acids [69]. Proteomic analysis of isolated LD fractions from SZ95 sebocytes revealed high levels of PLIN2 and PLIN3 and lipid metabolizing enzymes [36]. Moreover, SZ95 sebocytes markedly generated LDs when treated with linoleic acids, whereas silencing of PLIN3 attenuated linoleic acid-induced LD formation in these cells [70]. It was also reported that PLIN3 is expressed by vitamin A-storing stellate cells in the liver [71], and PLIN3 was induced in exercising human muscle cells [72]. The mammalian target of rapamycin complex 2 (mTORC2) is a key player in insulin signaling following phosphorylation of Akt. Mice lacking mTORC2 activity in skeletal muscle showed increased fat mas, and PLIN3, but not PLIN2 and PLIN5, was induced in these muscle cells. Finally, overexpression of PLIN3 in C2C12 myotubes increased TG stores in these cells [73]. Taken together, these data suggest that PLIN3 is a regulator of TG content in muscle cells.

### PLIN4

PLIN4 is unique among the five PLIN family proteins, with a molecular weight of nearly three-fold that of other PLINs and lack of a PAT domain (Fig. 2). PLIN4 has multiple sequence similarities with PLIN1**,** it is selectively expressed in adipocytes, and it is present in LDs and the cytosol [17]. Although little is known of the biological functions of PLIN4, its presence in human adipocytes was demonstrated following differentiation from human mesenchymal stem cells (hMSCs), and PLIN4 was induced with PLIN1 during the middle to late stages of adipocyte differentiation [33]. Moreover, co-induction of PLIN4 and PLIN1 during adipocyte differentiation was confirmed in 3T3-L1 cells and in OP9 mouse preadipocytes [74]. PLIN2 expression was also increased transiently during early stages of human adipocyte differentiation, but was replaced by PLIN1 and PLIN4 in later stages [33]. Thus, transient expression of PLIN proteins during human adipocyte maturation is very similar to the process described in murine 3T3-L1 cells [8].

The anti-psychotic medicine olanzapine is known to promote weight gain as one of its side effects [75]. Oral administration of olanzapine to rats for 5 weeks resulted in increased body weights and adipocyte size, and impaired lipolytic activity [76]. We examined the effect of olanzapine on adipocyte differentiation from hMSCs, and showed increased TG content and PLIN4 expression [33]. Olanzapine enhanced PLIN2 expression in the early phases of hMSC differentiation and increased PLIN4 levels in later stages. However, whereas LDs were enlarged under these conditions, most PLIN4 was distributed in the cytosol. These data suggest PLIN4 has roles in LD formation in human adipocytes that are distinct from those of PLIN1.

### PLIN5

Soon after the three LD-associated proteins containing PAT domain were recognized as belonging to the PAT family, another PAT domain protein with a unique tissue distribution pattern, initially named myocardial LD protein (MLDP), was discovered in BLAST searches [18]. This protein was concurrently identified as an LD protein in muscle tissues and was given other names (OXPAT, LSDP5), but it was ultimately named PLIN5 [11]. PLIN5 mRNA and protein are highly expressed in the heart tissue and they are regulated by PPARα. PLIN5 is also reportedly expressed in pancreatic islet β-cells and hepatic stellate cells [77, 78].

PLIN5-containing LDs are uniquely small particles and electron micrography experiments showed that these LD particles are associated with mitochondria in cardiac muscle cells [79, 80]. Specifically, PLIN5 was recovered in mitochondrial fractions from cardiomyocytes [79].

Osumi and colleagues established PLIN5-knockout (KO) mice and reported a central role for PLIN5 in the provision of energy for mitochondria in muscle cells [80]. In this study, LDs were undetectable in cardiac muscles from PLIN5-KO mice, even under high magnification electron micrographs. However, some LDs were detected in myotubes of soleus muscles after fasting. PLIN5-KO mice were physically normal but were intolerant to endurance exercise. Moreover, fatty acid oxidation was greater in cultured cardiomyocytes from PLIN5-KO mice than in those from wild-type mice. Because the primary energy source for cardiac muscles is fatty acid β-oxidation, these data suggest that muscle LDs play roles as reservoirs and distributors of fatty acids. It is noteworthy that PLIN2 is still present in lipid-poor particulates in cardiomyocytes when PLIN5 is depleted.

Regulation of lipid metabolism by PLIN5 was demonstrated in a murine islet β-cell cell line (MIN6 cells) expressing PLIN5 under the control of an adenovirus vector, and enhanced fatty acid incorporation into TG was associated with higher lipolysis rates in these cells [77]. In addition, PLIN5-expressing cells exhibited enhanced glucose-stimulated insulin secretion in the presence of fatty acids or 8-bromo-cAMP. Glucose tolerance was improved when PLIN5 was increased in mouse β-cells *in vivo*.

When hepatic stellate cells were changed to fibrotic phenotype in vitro, *Plin5* expression decreased while procollagen mRNA greatly increased. Transduction of the cells with PLIN5 increased cellular LD and inhibited fibrotic changes [78].

In a study by Chen et al., PLIN4-KO mice exhibited no phenotypic changes in adipose tissues, but had markedly reduced TG contents in heart tissues, potentially reflecting reduced expression of PLIN5 [81]. The murine genes for PLIN4 and PLIN5 are located on chromosome 17, and the *Plin4* gene begins only 1.7 kb downstream of *Plin5*. Hence, the peroxisome proliferation-responsive element (PPRE) in the *Plin5* promoter may also reside in the *Plin4* gene ORF region. Similarly, the human genes for PLIN4 and PLIN5 are closely located on chromosome 19, suggesting potential transcriptional interference between *Plin4* and *Plin5* genes.

## Proteins on LDs in steroidogenic cells

As described above, multiple studies have shown the presence of five PLIN proteins as major LD-associated proteins in various tissues and cell types, and some studies clearly demonstrated that PLIN proteins are crucial for the generation of LD particles. However, LDs that accumulate cholesterol in steroid hormone-producing cells may differ from TG-accumulating LDs [82]. PLIN1 and PLIN2 were identified in MA-10 Lydig cells and Y-1 adrenocortical cells in early studies [8, 83], and Hsieh et al. showed that different PLIN proteins were induced in mouse adrenal cortical cells and liver cells when treated with oleic acid or cholesterol. PLIN4 and PLIN1c, which is a splice variant of PLIN1, were present during the formation of CE-rich LDs [84]. It has been shown that CE-rich LDs behave differently from TG-rich LDs in Y-1 cells [85].

Mouse MLTC-1 Leydig tumor cells were originally derived from mouse Leydig cells that produced androgens from cholesterol, and they were shown to harbor medium sized LDs containing CE and TG. The protein profile of the LD fraction from MLTC-1 cells was distinct from those in macrophage and liver cell lines [86]. In further experiments, more than 50 proteins were identified in the LD fraction using LC-MS/MS analysis, and among these, the two enzymes 3β-hydroxysteroid dehydrogenase 1 (3β-HSD1) and 17β-HSD11 were determined to be the major proteins in this LD fraction, judging by the SDS-PAGE pattern. PLIN1, PLIN2, and PLIN3 together with ATGL were found in the LD fraction, but were present at lower levels than the major two enzymes. Following stimulation of MLTC-1 cells with luteinizing hormone, LDs became smaller and distributions of the LD proteins 3β-HSD1, 17β-HSD11, and PLIN1 changed from the buoyant fraction to intermediate density fractions. Thus, because 3β-HSD1 and 17β-HSD11 are key enzymes for steroid hormone synthesis, LDs in Leydig cells may have roles in cholesterol storage and act as reservoirs of enzymes for steroidogenesis.

Protein profiles of LDs in steroidogenic primary rat granulosa cells were investigated following incubation with either HDL or fatty acids to induce cholesterol-enriched LDs or TG-enriched LDs, respectively. In this study, quantitative proteomic analyses of differentially expressed proteins were performed using isobaric tag labeling [87], and 3β-HSD1 was among 61 proteins with >2-fold expression in cholesterol-enriched LDs compared with TG-enriched LDs.

Hydroxysteroid dehydrogenases (HSDs) are a family of enzymes that oxidize sterols, and sterol metabolizing enzymes are thought to predominate in smooth ER and mitochondria. However, among published proteomic analyses of LD proteins, HSDs are frequently reported in LD fractions with 17β-HSD11 [1–5, 88, 89]. Moreover, Motojima et al. identified a putative LD-association motif in 17β-HSD11 [90]. These observations suggest that HSD proteins to some extent are present in the LD fraction, and some cooperative activity may exist among the ER, mitochondria and LDs during the course of sterol metabolism.

## Conclusion

PAT family proteins are known as LD-associated proteins and multiple studies have characterized the physiological roles of PLIN1 in TG metabolism in WAT. Recent studies also show characteristic features of PLIN proteins that are consistent with tissue-specific expression patterns and cooperative functional roles in LDs. In addition, HSD proteins may participate in the formation and function of cholesterol-enriched LDs.

## Abbreviations

ACAT: Acyl-CoA cholesterol acyltransferase
ADRP: Adipose differentiation-related protein
AGPAT: Acylglycerophosphate acyltransferase
ATGL: Adipose triacylglycerol lipase
CE: Cholesterol ester
CGI: Comparative gene identification
DG: Diacylglycerol
DGAT: Diacylglycerol acyltransferase
DHA: Docosahexaenoic acid
ER: Endoplasmic reticulum
FC: Free cholesterol
FFA: Free fatty acid
GPAT: Glycerophosphate acyltransferase
HDL: High-density lipoprotein
hMSC: Human mesenchymal stem cell
HSD: Hydroxysteroid dehydrogenase
HSL: Hormone sensitive lipase
KO: Knockout
LD: Lipid droplet
LPS: Lipopolysaccharide
lysoPA: Lysophosphatidic acid
M6PR: Mannose-6-phosphate receptor
MG: Monoacylglycerol
MLDP: Myocardial lipid droplet protein
MLTC: Mouse Leydig tumor cell
PA: Phosphatidic acid
PKA: cAMP-dependent protein kinase
PLIN: Perilipin
PPAR: Peroxisome proliferator-activated receptor
RXR: Retinoid X receptor
TG: Triacylglycerol
TIP47: Tail-interacting protein of 47 kDa
WAT: White adipose tissue
